# Comparison of cytosine base editors and development of the BEable-GPS database for targeting pathogenic SNVs

**DOI:** 10.1186/s13059-019-1839-4

**Published:** 2019-10-23

**Authors:** Ying Wang, Runze Gao, Jing Wu, Yi-Chun Xiong, Jia Wei, Sipin Zhang, Bei Yang, Jia Chen, Li Yang

**Affiliations:** 1 CAS Key Laboratory of Computational Biology, CAS-MPG Partner Institute for Computational Biology, Shanghai Institute of Nutrition and Health, Shanghai Institutes for Biological Sciences, University of Chinese Academy of Sciences, Chinese Academy of Sciences, Shanghai, 200031 China; 2 School of Life Science and Technology, ShanghaiTech University, Shanghai, 201210 China; 3 CAS Center for Excellence in Molecular Cell Science, Shanghai Institute of Biochemistry and Cell Biology, Chinese Academy of Sciences, Shanghai, 200031 China; 4 University of Chinese Academy of Sciences, Beijing, 100049 China; 5 Shanghai Institute for Advanced Immunochemical Studies, ShanghaiTech University, Shanghai, 201210 China

**Keywords:** Base editing, Base editor, CRISPR/Cas, Cytidine deaminase, Pathogenic mutation

## Abstract

A variety of base editors have been developed to achieve C-to-T editing in different genomic contexts. Here, we compare a panel of five base editors on their C-to-T editing efficiencies and product purity at commonly editable sites, including some human pathogenic C-to-T mutations. We further profile the accessibilities of 20 base editors to all possible pathogenic mutations in silico. Finally, we build the BEable-GPS (Base Editable prediction of Global Pathogenic SNVs) database for users to select proper base editors to model or correct disease-related mutations. The in vivo comparison and in silico profiling catalog the availability of base editors and their broad applications in biomedical studies.

## Background

**Fig. 1:**
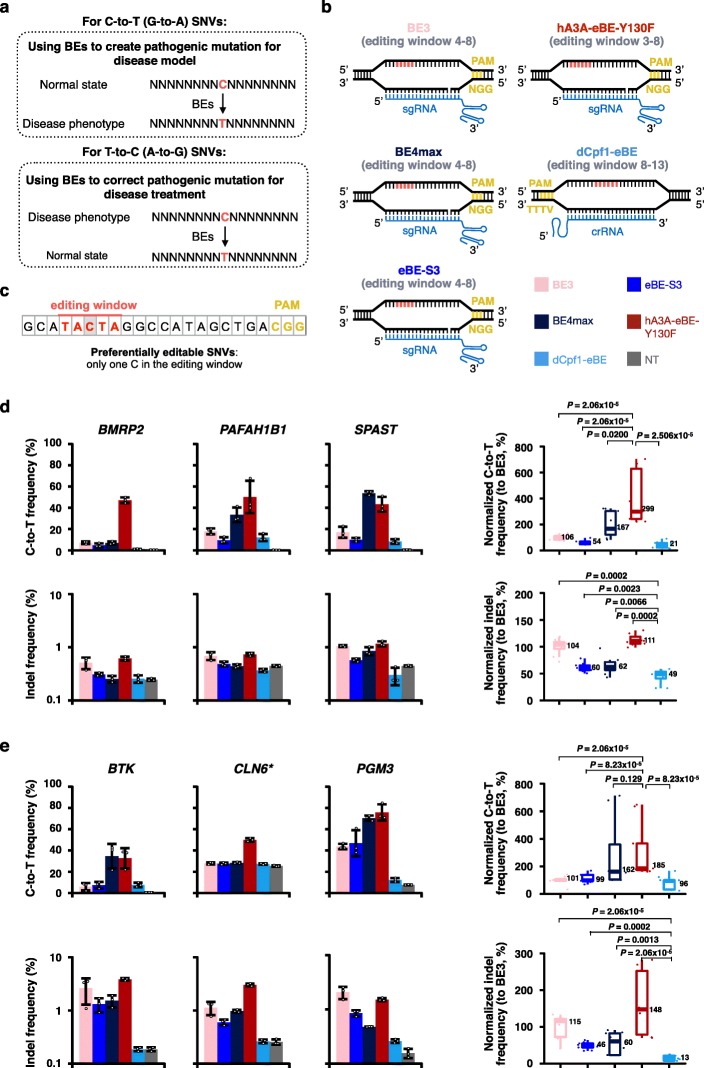
Comparison of base editing outcomes at pathogenic SNVs. **a** The diagram to illustrate the use of BEs in creating or correcting C-to-T (G-to-A) SNVs or T-to-C (A-to-G) SNVs to model or correct disease-related mutations. **b** The detailed target sites and editing windows of BE3, BE4max, eBE-S3, hA3A-eBE-Y130F, and dCpf1-eBE are shown. The cytosines were counted with the base distal to the PAM setting as position 1 in Cas9-based BEs and with the base proximal to the PAM setting as position 1 in Cpf1-based BEs. **c** The diagram to illustrate preferentially targetable SNVs. **d** Comparison of base editing outcomes at three pathogenic SNVs that can be created by five examined BEs in 293FT cells. **e** Comparison of base editing outcomes at three ABEmax-created pathogenic SNVs that can be corrected by five examined BEs in 293FT cells. The C-to-T editing (top) and indel (bottom) frequencies were individually shown at target sites in **d** and **e**. Asterisk, the T-to-C mutation created by ABEmax at the *CLN6* gene locus is heterozygous, indicated by the high basal level of C-to-T correction by non-transfected control in **e**. NT, non-transfected. Data are shown as mean ± s.d. from three independent experiments. Statistical analysis of normalized C-to-T editing frequencies and indel frequencies at these three pathogenic SNV sites is accordingly shown in the right panel in **d** and **e**. Setting the ones induced by BE3 as 100%. *P* value, one-tailed Wilcoxon rank sum test. The median and interquartile range (IQR) are shown

A number of base editors (BEs) [1–6], which combine different APOBEC (apolipoprotein B mRNA editing enzyme, catalytic polypeptide-like)/AID (activation-induced deaminase) cytidine deaminase family members [7, 8] with distinct CRISPR/Cas proteins [9, 10], have been developed to achieve programmable C-to-T changes in different sequence contexts or backgrounds. Distinct to Cas nucleases, which trigger homology-directed repair (HDR)-mediated gene correction by cleaving DNA double strands, BEs induce base changes in targeted genomic regions independent of the generation of DNA double-strand breaks (DSB) generally. Guided by the Cas moiety, BEs catalyze direct C-to-T changes with its fused cytidine deaminase moiety. A uracil DNA glycosylase inhibitor (UGI) is fused to BEs to prevent unintended mutagenesis during the process of base editing [1, 2], and additional UGIs co-expressed in *trans* with BEs (enhanced BE, eBE) further enhance the efficiency and fidelity of base editing [11]. BEs hold the potential to be used for correcting and creating pathogenic point mutations (Fig. 1a) [12–14]. However, BEs with different Cas proteins, e.g., Cas9 or Cas12a (also known as Cpf1), and different deaminases, e.g., rat APOBEC1 (rA1) or human APOBEC3A (hA3A), have not been directly compared for their utility in creating or correcting pathogenic point mutations. More importantly, a database comprehensively cataloging pathogenic point mutations that can be corrected or created by different BEs has been lacking. In this study, we experimentally compare a panel of five BEs for their editing efficiency and product purity at sites of human pathogenic C-to-T mutations that can be created or corrected by the same panel of BEs. We further profile the accessibilities of 20 BEs to all reported human pathogenic-related T-to-C or C-to-T point mutations in silico and build a BEable-GPS (Base Editable prediction of Global Pathogenic SNVs) database to provide a resource for potential gene therapies and biomedical studies.

## Results and discussion

The combination of different cytidine deaminases with distinct Cas proteins extends the scope of base editing in different sequence contexts or backgrounds; however, it also results in variable targeting preferences, which hampers the direct comparison of BEs. To solve this problem, we selected five BEs, including BE3 [1], eBE-S3 [11], BE4max [15], hA3A-eBE-Y130F [6], and dCpf1-eBE [5], to compare their base editing efficiency and product purity at the same genomic target sites. These selected five BEs have similar widths of editing window (~ 5 bp) for comparison (Fig. 1b).

At three previously reported target sites [5] that can be edited by all five BEs, BE4max and hA3A-eBE-Y130F induced higher C-to-T editing frequencies than the other examined BEs in 293FT cells (Additional file 1: Figure S1a, b), while hA3A-eBE-Y130F also exhibited slightly higher indel frequencies (Additional file 1: Figure S1a, c). The relatively high indels induced by hA3A-eBE-Y130F are likely caused by the high cytidine deamination activity of its hA3A moiety [16, 17]. Although showing the lowest editing frequencies among all five tested BEs, dCpf1-eBE induced fewer indels and non-C-to-T conversions than the other BEs did (Additional file 1: Figure S1a, c) and therefore yielded purer editing products (Additional file 1: Figure S1d). Assumedly, the catalytically dead Cpf1 moiety in dCpf1-eBE makes its low editing frequency but high product purity (Additional files 3, 4, 5, and 6).

We next sought to compare the performance of these BEs to create human pathogenic C-to-T SNVs. Among reported pathogenic C-to-T SNVs [18], we selected three sites, at which all five BEs have overlapping editing windows (Additional file 1: Figure S2a). Importantly, the cytosine in each of the three selected sites is the only cytosine in the editing window, referred to as preferentially editable SNVs (Fig. 1c). Theoretically, the C-to-T conversions at these three target sites could be used to mimic human genetic disorders (Additional file 1: Figure S2b). At these sites, BE4max and hA3A-eBE-Y130F also induced higher levels of editing frequencies than the other examined BEs in 293FT cells (Fig. 1d, top), consistent with the results obtained at non-pathogenic target sites (Additional file 1: Figure S1). Notably, only hA3A-eBE-Y130F yielded efficient base editing at the loci of *BMRP2* (Fig. 1d, top), while no obvious editing was induced by the other BEs. The indel frequencies induced by dCpf1-eBE were lower than those induced by the other BEs (Fig. 1d, bottom). Meanwhile, the C-to-T fraction induced by dCpf1-eBE was significantly higher than those by the other BEs (Additional file 1: Figure S2c), showing that dCpf1-eBE yielded purer editing products.

Another important application of BEs is to correct pathogenic mutations, which theoretically could be used in pre-clinic or clinic studies [19]. To test base editing efficiency and precision in correcting pathogenic mutations of these BEs, we took advantage of ABEmax [15] to first create T-to-C mutations and then to correct them by the aforementioned five BEs (Additional file 1: Figure S3). Three reported pathogenic T-to-C/A-to-G SNV sites that can be preferentially corrected by all five BEs were selected for correction study (Additional file 1: Figure S3a, b). These pathogenic T-to-C/A-to-G mutations were generated by ABEmax individually in 293FT cells (Additional file 1: Figure S3c), and single-colony-derived cell lines with corresponding T-to-C mutations were further confirmed by Sanger sequencing (Additional file 1: Figure S3d). These T-to-C/A-to-G mutations that mimic pathogenic SNVs were further corrected by five tested BEs. As shown in Fig. 1e (top), BE4max and hA3A-eBE-Y130F induced higher efficiencies than the other examined BEs. Notably, only hA3A-eBE-Y130F yielded efficient base editing at the loci of *CLN6* (Fig. 1e, top), while the others induced editing similar to the background level. As expected, dCpf1-eBE induced purer editing products than the other BEs though it induced low levels of C-to-T correction efficiency (Fig. 1e, bottom and Additional file 1: Figure S4).

We further compared three representative BEs, including hA3A-eBE-Y130F with the highest editing efficiency, dCpf1-eBE with the purest editing product, and eBE-S3 with intermediate editing efficiency and product purity (Fig. 1d, e and Additional file 1: Figure S1), at additional sites for their editing efficiencies and product purities. Of note, these three selected BEs all express three extra copies of free UGI to enhance editing performance. As expected, hA3A-eBE-Y130F induced the highest editing frequency and dCpf1-eBE yielded the purest C-to-T editing product (Additional file 1: Figure S5, S6), at eight genomic target sites (Additional file 1: Figure S5, including three sites that have been examined with five tested BEs in Additional file 1: Figure S1) as well as eight target sites where C-to-T conversions create pathogenic SNVs (Additional file 1: Figure S6, including three sites that have been examined with five tested BEs in Additional file 1: Figure S2). Meanwhile, we also compared these three representative BEs at the same sites in another human cell line U2OS and obtained similar results (Additional file 1: Figures S7, S8).

**Fig. 2:**
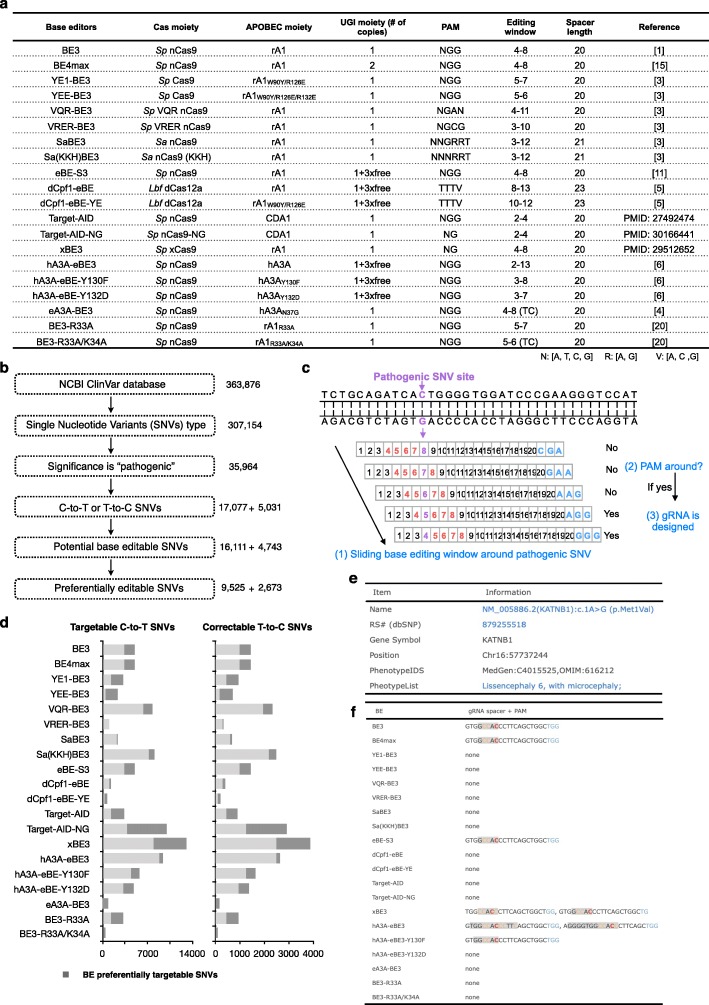
In silico base editable landscape of pathogenic SNVs. **a** Summary of 20 selected BEs used in BEable-GPS. PAM, editing window, spacer length, and reference are listed. **b** The pipeline for filtering BE editable pathogenic SNVs from NCBI ClinVar database. The numbers of variants are listed in the right for each filtering step. **c** The workflow of judging whether a pathogenic SNV is potentially targetable. If there are nearby PAM sequences when a SNV is in the editing window, this SNV is considered to be a potentially targetable site by BEs. **d** Statistics of number distribution of targetable or preferentially targetable pathogenic SNVs by each BE summarized in **a**. **e** The information of one representative pathogenic SNV is provided in the BEable-GPS online website. **f** The gRNA spacer region and PAM sequence of each BE for one representative SNV are shown. Pathogenic SNV in red, bystander editing site in yellow, editing window in gray, and PAM in blue

As BEs can be used to introduce base substitutions to mimic or revert the pathogenic SNVs (Fig. 1), we set up to computationally profile all human pathogenic C-to-T or T-to-C SNVs to determine which types of BEs might be more suitable for creating or correcting mutations. Twenty BEs with different PAM sequences and editing windows, including the five aforementioned ones, were used for this in silico analysis. The PAM sequences and editing windows of these 20 BEs are listed in Fig. 2a.

For all pathogenic SNVs reported in the NCBI ClinVar database (Fig. 2b), we searched their flanking regions to find nearby PAM sequences that could fit the pathogenic SNV into the editing windows of examined BEs. Based on the existence of PAM sequences, we predicted whether a given SNV could be potentially edited by a specific BE (Fig. 2c). With 20 analyzed BEs, about 94.34% of 17,077 pathogenic C-to-T SNVs could be generated by at least one BE to model the relevant genetic disorders and 94.28% of 5031 pathogenic T-to-C SNVs could be corrected by at least one BE to examine the potential therapeutic effects. The potentially editable SNVs are summarized in Fig. 2d. The in silico profiling of base editable pathogenic SNVs thus suggests broad applications of BEs for human disease study and potential treatment.

To conveniently access the information of these base editable pathogenic point mutations, we constructed a BEable-GPS (http://www.picb.ac.cn/rnomics/BEable-GPS) database for annotation. A “search” function is available to query pathogenic SNVs according to gene symbols, genomic locations or disease phenotypes, and their accessibilities to different BEs (Additional file 1: Figure S9a). With selected BEs, all targetable pathogenic SNVs in queried locations or disease phenotypes can be retrieved in the output list (Additional file 1: Figure S9b). By clicking “Link” button next to a selected SNV, its name (NCBI ClinVar ID), related dbSNP number, chromosome position, gene symbol, related phenotype ID (Fig. 2e), and designed gRNA spacer sequences with the corresponding PAMs highlighted for all applicable BEs (Fig. 2f) are available for further survey.

An online “analysis” function is also available to design specific gRNAs for editable cytosines/guanines from any input sequence (Additional file 1: Figure S10a). Of note, users can also define a specific PAM sequence, editing window, and spacer length to find specific base editable sites for further analysis (Additional file 1: Figure S10a, bottom). All cytosines or guanines that are targetable by the analyzed BEs will be listed together with specific gRNA spacer sequences (Additional file 1: Figure S10b). This online “analysis” function thus expands the application of the BEable-GPS database from pathogenic SNV sites to almost all editable cytosines and guanines. For both search and analysis functions, users can select the union or the intersection of these 20 analyzed BEs for survey and comparison (Additional file 1: Figures S9a, S10a).

It will be of interest for researchers to access BEable-GPS and embedded toolsets for their experimental designs to model or correct disease-related mutations. Of note, to reduce substantial off-target mutations, engineered BEs have been continuously developed for precise base editing [20]. We will keep updating this database by including more BEs to provide additional choices for the study of pathogenic mutations and by incorporating off-target prediction to suggest cautions in the future.

## Methods

### Cell culture and transfection

293FT and U2OS cells from ATCC were tested to exclude mycoplasma contamination and not authenticated. For base editing in genomic DNA, 293FT and U2OS cells were seeded in a 24-well plate at a density of 1 × 10^5^ cells/well and transfected with 250 μl serum-free Opti-MEM containing 2.52 μl Lipofectamine LTX (Invitrogen/Life Technologies), 0.84 μl Lipofectamine Plus (Invitrogen/Life Technologies), 0.5 μg BE expression vector (BE3, eBE-S3, BE4max, hA3A-eBE-Y130F or dCpf1-eBE, respectively), and 0.34 μg crRNA or sgRNA-expressing plasmid. After 72 h, the genomic DNA was extracted from the cells with QuickExtract DNA Extraction Solution for subsequent analyses.

To generate T-to-C/A-to-G mutations that mimic pathogenic SNV sites individually at the *BTK*,*CLN6*, and *PGM3* loci, 293FT cells were seeded into a six-well plate at a density of 3 × 10^5^ cells per well and transfected with 250 μl serum-free Opti-MEM containing 7.56 μl Lipofectamine LTX (Invitrogen/Life Technologies), 2.52 μl Lipofectamine Plus (Invitrogen/Life Technologies), 1.5 μg ABEmax, and 1.02 μg sgRNA-expressing plasmid (sgBTK, sgCLN6 or sgPGM3, respectively). The genomic DNAs of single-cell colonies were individually purified, and ABEmax-created T-to-C mutations were validated by Sanger sequencing (Additional file 2).

### Targeted DNA sequencing and data analysis

Targeted genomic sites were PCR amplified, and an indexed DNA library was prepared for deep sequencing. Indel frequencies were calculated by dividing reads containing at least one inserted and/or deleted nucleotide by all the mapped reads at the same region. Base substitution frequencies were calculated by dividing base substitution reads by total reads.

### BE editable analysis of pathogenic SNVs

The pathogenic mutation sites were downloaded from the NCBI ClinVar database. “Single-nucleotide variants (SNVs)” of “pathogenic” significance were extracted for further analysis. The SNV names including “C>T” or “G>A” were identified as pathogenic C-to-T/G-to-A SNVs. The SNV names including “T>C” or “A>G” were identified as pathogenic T-to-C/A-to-G SNVs. The flanking sequence (30 nucleotides upstream and downstream of the SNV site) was extracted from genome sequence according to the coordinate (GRCh38) of SNVs for targetable analysis.

### Statistical analysis

*P* values were calculated from one-tailed Wilcoxon rank sum test in this study.

## Supplementary information

**Additional file 1:** **Figure S1.** Comparison of base editors at overlapped target sites in 293FT cells. **Figure S2.** Three pathogenic SNVs that can be created by BEs in 293FT cells. **Figure S3.** Three T-to-C mutations are created by ABEmax in 293FT cells to mimic pathogenic T-to-C/A-to-G SNVs. **Figure S4.** Comparison of product purity at three ABEmax-generated T-to-C mutations that can be corrected by BEs in 293FT cells. **Figure S5.** Comparison of base editing outcomes at eight overlapped target sites in 293FT cells. **Figure S6.** Comparison of base editing outcomes at eight pathogenic SNVs in 293FT cells. **Figure S7.** Comparison of base editing outcomes at eight overlapped target sites in U2OS cells. **Figure S8.** Comparison of base editing outcomes at eight pathogenic SNVs in U2OS cells. **Figure S9.** Construction of BEable-GPS website for base editable pathogenic SNVs. **Figure S10.** Function of gRNA design embedded in the BEable-GPS website.

**Additional file 2:** **Table S1.** Oligos used for CBE-gRNA-expressing plasmid construction. **Table S2.** Oligos used for ABEmax-gRNA-expressing plasmid construction. **Table S3.** gRNA target sequences and PCR primers for amplifying genomic DNA.

**Additional file 3:** **Table S4.** Calculation of indels of base editing results in Fig. 1 and Additional file 1: Figures S1–S4.

**Additional file 4:** **Table S5.** Calculation of base substitutions of base editing results in Fig. 1 and Additional file 1: Figures S1–S4.

**Additional file 5:** **Table S6.** Calculation of indels of base editing results in Additional file 1: Figures S5–S8.

**Additional file 6:** **Table S7.** Calculation of base substitutions of base editing results in Additional file 1: Figure S5–S8.

**Additional file 7.** Review history.

## Data Availability

BEable-GPS is available at http://www.picb.ac.cn/rnomics/BEable-GPS. The core source code of BEable-GPS used for gRNA design for pathogenic SNVs are freely available under the MIT license at https://github.com/suduwoniu/BEable-GPS [21], and an archival version of this code is available on Zenodo with DOI 10.5281/zenodo.3460965 [22]. Targeted deep sequencing datasets are available at the NCBI Gene Expression Omnibus with accession code GSE136749 (https://www.ncbi.nlm.nih.gov/geo/query/acc.cgi?acc= GSE136749) [23] and National Omics Data Encyclopedia with access number NODE: OEP000459 (https://www.biosino.org/node/project/detail/OEP000459) [24].
