# Supplementary material for: Comparison of cytosine base editors and development of the BEable-GPS database for targeting pathogenic SNVs
Source: Genome Biol. 2019 Oct 23;20:218. doi: 10.1186/s13059-019-1839-4 (PMC6806563; doi:10.1186/s13059-019-1839-4)
Supplement: Supplementary file 1 — Additional file 1: Figure S1. Comparison of base editors at overlapped target sites in 293FT cells. Figure S2. Three pathogenic SNVs that can be created by BEs in 293FT cells. Figure S3. Three T-to-C mutations are created by ABEmax in 293FT cells to mimic pathogenic T-to-C/A-to-G SNVs. Figure S4. Comparison of product purity at three ABEmax-generated T-to-C mutations that can be corrected by BEs in 293FT cells. Figure S5. Comparison of base editing outcomes at eight overlapped target sites in 293FT cells. Figure S6. Comparison of base editing outcomes at eight pathogenic SNVs in 293FT cells. Figure S7. Comparison of base editing outcomes at eight overlapped target sites in U2OS cells. Figure S8. Comparison of base editing outcomes at eight pathogenic SNVs in U2OS cells. Figure S9. Construction of BEable-GPS website for base editable pathogenic SNVs. Figure S10. Function of gRNA design embedded in the BEable-GPS website. [file 13059_2019_1839_MOESM1_ESM.docx]

**Supplementary figures**

**Figure S1. Comparison of base editors at overlapped target sites in 293FT cells. a** Comparison of base editing outcomes at three reported target sites mediated by five BEs. The C-to-T editing frequencies of indicated cytosines, the indel frequencies and the fractions of cytosine substitutions (targeted sites with a C-to-T editing efficiency less than 5% were excluded, indicated as N/A) were individually shown at the indicated genomic target sites under different BE treatment. The target-site sequences, intersected editing cytosines of five BEs are shown in the left. NT, non-transfected. Data are shown as mean ± s.d. from three independent experiments. **b** Statistical analysis of normalized C-to-T editing efficiencies at three target sites in (a). **c** Statistical analysis of indel frequencies at three target sites in (a). **d** Statistical analysis of normalized C-to-T substitution fractions at three target sites in (a). Setting the ones induced by BE3 as 100%. *P* value, one-tailed Wilcoxon rank sum test. The median and interquartile range (IQR) are shown.

**
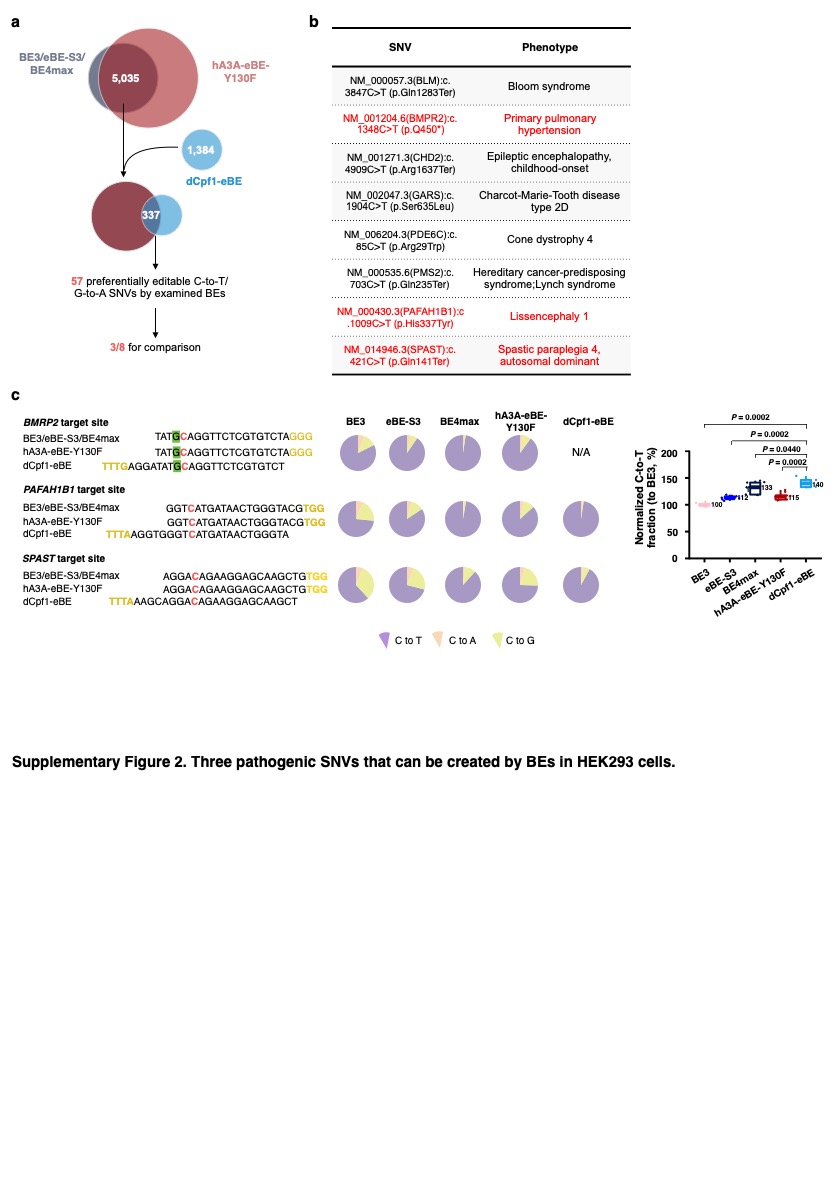
**

**Figure S2. Three pathogenic SNVs that can be created by BEs in 293FT cells.** **a** The number of editable and preferentially editable pathogenic C-to-T SNVs jointly targetable by five BEs are shown. **b** Eight preferentially editable SNVs that can be created by five BEs are selected for experimental analysis, of which three SNVs (in red) are for the comparison of five BEs. Pathogenic SNVs, corresponding amino acid changes and related diseases are shown. **c** Comparison of cytosine substitutions at three pathogenic SNVs that can be created by five BEs in 293FT cells. Targeted sites with a C-to-T editing efficiency less than 5% were excluded, indicated as N/A. The target-site sequences, intersected editing cytosines of five BEs are shown in the left. The guanines in green indicating the targetable cytosines are in a CpG or GpC context. **d** Statistical analysis of normalized C-to-T substitution fractions at these three pathogenic SNVs in (c). Setting the ones induced by BE3 as 100%. *P* value, one-tailed Wilcoxon rank sum test. The median and interquartile range (IQR) are shown.

**
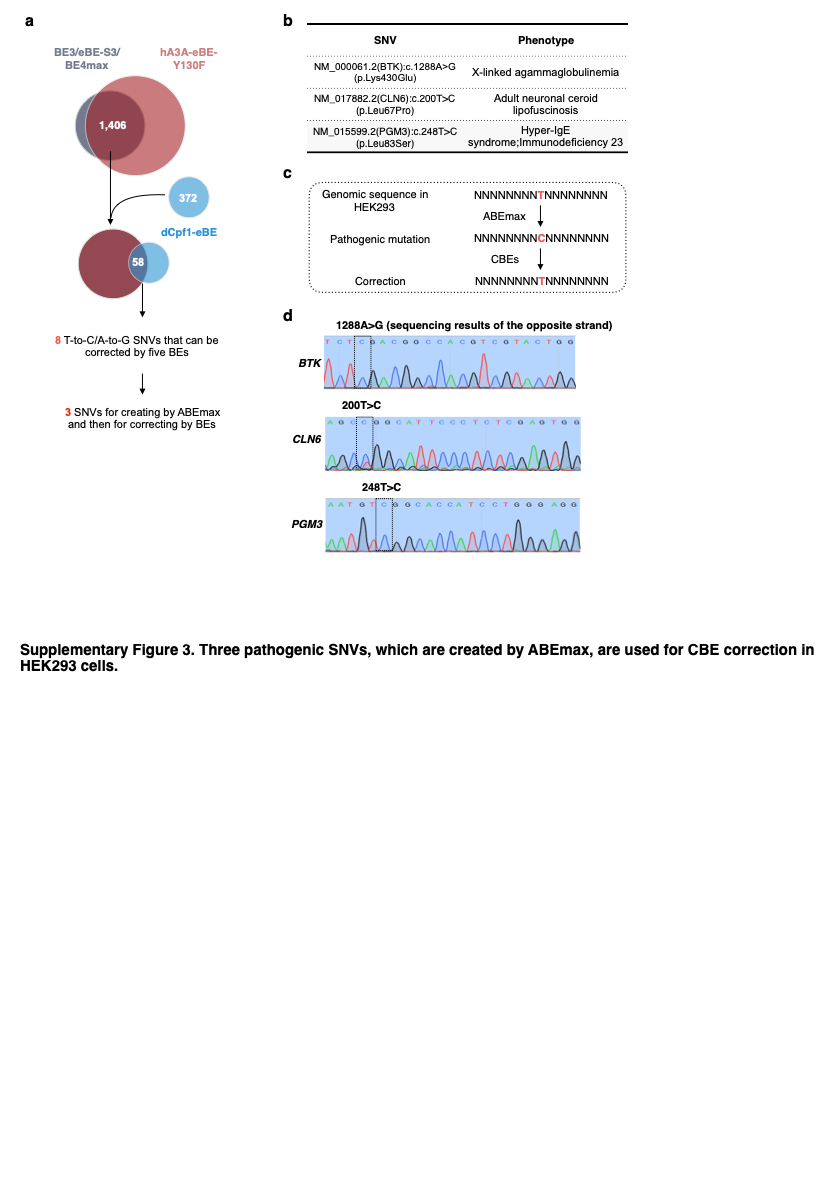
**

**Figure S3. Three T-to-C mutations are created by ABEmax in 293FT cells to mimic pathogenic T-to-C/A-to-G SNVs.** **a** The number of pathogenic T-to-C/A-to-G SNVs, which can be jointly targeted by five BEs, are shown. **b** Three T-to-C (or A-to-G) SNVs are selected to be used for the comparison of five examined BEs in their activities of C-to-T correction. Pathogenic SNVs, corresponding amino acid changes and related diseases are shown. **c** The diagram to illustrate the design of using ABEmax to create pathogenic T-to-C (or A-to-G) SNVs and then using CBEs to correct them. **d** Sanger sequencing to confirm corresponding T-to-C/A-to-G mutations created by ABEmax individually in single-colony-derived cell lines (b).

**Figure S4. Comparison of product purity at three ABEmax-generated T-to-C mutations that can be corrected by BEs in 293FT cells. a** Fractions of cytosine substitution at three T-to-C/A-to-G pathogenic SNV mutations created by ABEmax in 293FT cells (targeted sites with a C-to-T editing efficiency less than 5% were excluded, indicated as N/A). The target-site sequences, intersected editing cytosines of the five BEs are shown in left. The guanines in green indicating the targetable cytosines are in a CpG or GpC context. **b** Statistical analysis of normalized C-to-T substitution fractions at these three T-to-C/A-to-G pathogenic SNV mutations created by ABEmax in (a). Setting the ones induced by BE3 as 100%. *P* value, one-tailed Wilcoxon rank sum test. The median and interquartile range (IQR) are shown.

**Figure S5. Comparison of base editing outcomes at eight overlapped target sites in 293FT cells**. **a** Comparison of base editing outcomes at eight reported target sites mediated by three BEs. The C-to-T editing frequencies of indicated cytosines, the indel frequencies and the fractions of cytosine substitutions (targeted sites with a C-to-T editing efficiency less than 5% were excluded, indicated as N/A) were individually shown at the indicated genomic target sites under different BE treatment. The target-site sequences, intersected editing cytosines of three BEs are shown in the left. NT, non-transfected. Asterisk denotes an unusually high basal indel frequency (or amplification, sequencing or alignment artifact) at the examined *RUNX1* site. Data are shown as mean ± s.d. from three independent experiments. **b** Statistical analysis of normalized C-to-T editing efficiencies at eight target sites in (a). **c** Statistical analysis of indel frequencies at eight target sites in (a). **d** Statistical analysis of normalized C-to-T substitution fractions at eight target sites in (a). Setting the ones induced by eBE-S3 as 100%. *P* value, one-tailed Wilcoxon rank sum test. The median and interquartile range (IQR) are shown.

**Figure S6. Comparison of base editing outcomes at eight pathogenic SNVs in 293FT cells**. **a** Comparison of base editing outcomes at eight pathogenic sites mediated by three BEs. The C-to-T editing frequencies of indicated cytosines, the indel frequencies and the fractions of cytosine substitutions (targeted sites with a C-to-T editing efficiency less than 5% were excluded, indicated as N/A) were individually shown at the indicated target sites under different BE treatment. The target-site sequences, intersected editing cytosines of three BEs are shown in the left. The guanines in green indicating the targetable cytosines are in a CpG or GpC context. NT, non-transfected. Asterisk denotes an unusually high basal indel frequency (or amplification, sequencing or alignment artifact) at the examined *CHD2* site. Data are shown as mean ± s.d. from three independent experiments. **b** Statistical analysis of normalized C-to-T editing efficiencies at eight pathogenic SNVs in (a). **c** Statistical analysis of indel frequencies at eight pathogenic SNVs in (a). **d** Statistical analysis of normalized C-to-T substitution fractions at eight pathogenic SNVs in (a). Setting the ones induced by eBE-S3 as 100%. *P* value, one-tailed Wilcoxon rank sum test. The median and interquartile range (IQR) are shown.

**Figure S7. Comparison of base editing outcomes at eight overlapped target sites in U2OS cells**. **a** Comparison of base editing outcomes at eight reported target sites mediated by three BEs. The C-to-T editing frequencies of indicated cytosines, the indel frequencies and the fractions of cytosine substitutions (targeted sites with a C-to-T editing efficiency less than 5% were excluded, indicated as N/A) were individually shown at the indicated genomic target sites under different BE treatment. The target-site sequences, intersected editing cytosines of three BEs are shown in the left. NT, non-transfected. Asterisk denotes an unusually high basal indel frequency (or amplification, sequencing or alignment artifact) at the examined *RUNX1* site. Data are shown as mean ± s.d. from three independent experiments. **b** Statistical analysis of normalized C-to-T editing efficiencies at eight target sites in (a). **c** Statistical analysis of indel frequencies at eight target sites in (a). **d** Statistical analysis of normalized C-to-T substitution fractions at eight target sites in (a). Setting the ones induced by eBE-S3 as 100%. *P* value, one-tailed Wilcoxon rank sum test. The median and interquartile range (IQR) are shown.

**Figure S8. Comparison of base editing outcomes at eight pathogenic SNVs in U2OS cells**. **a** Comparison of base editing outcomes at eight pathogenic sites mediated by three BEs. The C-to-T editing frequencies of indicated cytosines, the indel frequencies and the fractions of cytosine substitutions (targeted sites with a C-to-T editing efficiency less than 5% were excluded, indicated as N/A) were individually shown at the indicated target sites under different BE treatment. The target-site sequences, intersected editing cytosines of three BEs are shown in the left. The guanines in green indicating the targetable cytosines are in a CpG or GpC context. NT, non-transfected. Asterisk denotes an unusually high basal indel frequency (or amplification, sequencing or alignment artifact) at the examined *CHD2* site. Data are shown as mean ± s.d. from three independent experiments. **b** Statistical analysis of normalized C-to-T editing efficiencies at eight pathogenic SNVs in (a). **c** Statistical analysis of indel frequencies at eight pathogenic SNVs in (a). **d** Statistical analysis of normalized C-to-T substitution fractions at eight pathogenic SNVs in (a). Setting the ones induced by eBE-S3 as 100%. *P* value, one-tailed Wilcoxon rank sum test. The median and interquartile range (IQR) are shown.

**Figure S9. Construction of BEable-GPS website for base editable pathogenic SNVs. a** The webpage for inquiring pathogenic SNVs according to gene symbol/genomic region/disease phenotype and BE options. For comparing BEs, “intersection” should be chosen to inquire intersected pathogenic SNVs targeted by selected BEs. **b** The inquiring results are listed in a new webpage. SNV related diseases were shown. Clicking “Link” button will get into a new webpage showing detailed SNV information (Figs. 2e, f).

**Figure S10. Function of gRNA design embedded in the BEable-GPS website. a** The webpage for editable analysis of one input sequence. The editing range could be friendly changed to whatever you are interested in. User defined BEs are supported by inputting PAM sequence, PAM type, editing window and spacer length. **b** Editable analysis results are shown. All possible editable “C”s with their relative positions in sequence are shown.
