# Supplementary material for: Comparison of cytosine base editors and development of the BEable-GPS database for targeting pathogenic SNVs
Source: Genome Biol. 2019 Oct 23;20:218. doi: 10.1186/s13059-019-1839-4 (PMC6806563; doi:10.1186/s13059-019-1839-4)
Supplement: Supplementary file 2 — Additional file 2: Table S1. Oligos used for CBE-gRNA-expressing plasmid construction. Table S2. Oligos used for ABEmax-gRNA-expressing plasmid construction. Table S3. gRNA target sequences and PCR primers for amplifying genomic DNA. [file 13059_2019_1839_MOESM2_ESM.docx]

**Supplementary methods**

**Plasmid construction**

Oligonucleotides hBLM_cpf1_sg1_FOR/hBLM_cpf1_sg1_REV were annealed and ligated into BsaI-linearized pLb-Cpf1-pGL3-U6-sgRNA to generate the crBLM expression vector pcrBLM. Oligonucleotides hBLM_sp_sg1_FOR/hBLM_sp_sg1_REV were annealed and ligated into BsaI-linearized pGL3-U6-sgRNA-PGK-puromycin to generate the sgBLM expression vector psgBLM. Other crRNA and sgRNA expression vectors were constructed in the same manner. Sequences of the oligonucleotides used for CBE-gRNA-expressing plasmid construction are listed in Table S1 and sequences of the oligonucleotides used for ABEmax-gRNA-expressing plasmid construction are listed in Table S2.

**Cell culture and transfection**

293FT and U2OS cells from ATCC were maintained in DMEM (10566, Gibco/Thermo Fisher Scientific) with 10% FBS (16000-044, Gibco/Thermo Fisher Scientific) and were tested to exclude mycoplasma contamination.

For base editing in genomic DNA, 293FT and U2OS cells were seeded in a 24-well plate at a density of 1×10^5^ cells/well and transfected with 250μl serum-free Opti-MEM containing 2.52μl Lipofectamine LTX (Invitrogen/LifeTechnologies), 0.84μl Lipofectamine Plus (Invitrogen/Life Technologies), 0.5μgBE expression vector (BE3, eBE-S3, BE4max, hA3A-eBE-Y130F or dCpf1-eBE, respectively) and 0.34μg crRNA or sgRNA-expressing plasmid. After 72 h, the genomic DNA was extracted from the cells with QuickExtractDNA Extraction Solution (QE09050, Epicentre).

To generate T-to-C/A-to-G mutations that mimic pathogenic SNV sites individually at the *BTK*,*CLN6* and *PGM3*loci, 293FT cells were seeded into a six-well plate at a density of 3 × 10^5^ cells per well and transfected with 250μl serum-free Opti-MEM containing 7.56μl Lipofectamine LTX (Invitrogen/LifeTechnologies), 2.52μl Lipofectamine Plus (Invitrogen/Life Technologies), 1.5 μg ABEmax and 1.02μg sgRNA-expressing plasmid (sgBTK, sgCLN6 or sgPGM3, respectively). After 24h, puromycin was added at a concentration of 5μg/ml and with the treatment, transfected cells were kept in 48 h, then trypsinized and transferred into a 100-mm dish for the selection of single-cell colonies. The genomic DNAs of single-cell colonies were individually purified, and ABEmax-created T-to-C mutations were validated by Sanger sequencing. Of note, ABEmax also induced a bystander mutation (mutation other than the intended one in the editing window) at each of *BTK* and *CLN6* loci. These bystander mutations are not reported in NCBI ClinVar database and thus excluded from the subsequent analysis.

**DNA library preparation and sequencing**

Target genomic sites were PCR amplified by high-fidelity DNA polymerase PrimeSTAR HS (Clonetech) with primers flanking each examined sgRNA target site. The PCR primers used to amplify target genomic sequences are listed in Table S3. Indexed DNA libraries were prepared by using the TruSeqChIP Sample Preparation Kit (Illumina) with some minor modifications. Briefly, the PCR products amplified from genomic DNA regions were fragmented by Covaris S220 and then PCR amplified by using the TruSeqChIP Sample Preparation Kit (Illumina). After being quantitated with the Qubit High-Sensitivity DNA kit (Life, Invitrogen), PCR products with different tags were pooled together for deep sequencing using the Illumina NextSeq 500 (2×150, 1×150) at the CAS-MPG Partner Institute for Computational Biology Omics Core, Shanghai, China. Raw read qualities were evaluated by FastQC (v0.11.4). For paired-end sequencing, only R1 reads were used. Adaptor sequences and read sequences on both ends with Phred quality score lower than 28 were trimmed. Trimmed reads were then mapped with the BWA-MEM algorithm (BWA v0.7.9a) to target sequences. After being piled up with samtools (v0.1.18), indels and base substitutions were further calculated as below.

**Indel frequency calculation**

Indels were estimated in the aligned regions spanning from upstream eight nucleotides of the target site to downstream 19 nucleotides of PAM sites (50 bp). Indel frequencies were subsequently calculated by dividing reads containing at least one inserted and/or deleted nucleotide by all the mapped reads at the same region. Counts of indel-containing reads and total mapped reads are listed in Tables S4 and S6.

**Base substitution calculation**

Base substitutions were selected at each position of the examined gRNA target sites that mapped with at least 1,000 independent reads, and obvious base substitutions were only observed at the targeted base editing sites. Counts of reads for each base and total reads are listed in Tables S5 and S7. Base substitution frequencies were calculated by dividing base substitution reads by total reads.

**BE editable analysis of pathogenic SNVs**

The pathogenic mutation sites were downloaded from the NCBI ClinVar database. “Single Nucleotide Variants (SNVs)” of “pathogenic” significance were extracted for further analysis. The SNV name including “C>T” or “G>A” were identified as pathogenic C-to-T/G-to-A SNVs. The SNV name including “T>C” or “A>G” were identified as pathogenic T-to-C/A-to-G SNVs. The flanking sequence (30 nucleotides upstream and downstream of the SNV site) was extracted from genome sequence according to the coordinate (GRCh38) of SNVs for targetable analysis (Fig. 2b). An base editor editable pathogenic SNV site is selected with: 1) whether there is a nearby PAM sequence, and 2) whether this SNV fits in the editing window of examined BEs (Fig. 2c). If a pathogenic SNV locates in the editing window of examined BEs and with no other “C”s, this SNV was referred to as a preferentially targetable SNV (Fig. 1c).

**Statistical analysis**

*P* values were calculated from one-tailed Wilcoxon rank sum test in this study.

**SUPPLEMENTARY TABLES**

**Supplementary Table 1: Oligos used for CBE-gRNA-expressing plasmid construction.**

| Primer name | Sequence (5' to 3') |
| --- | --- |
| hBLM_cpf1_sg1_FOR | AGATAGTATTACAGAAATACTCTGAAT |
| hBLM_cpf1_sg1_REV | AAAAATTCAGAGTATTTCTGTAATACT |
| hBLM_sp_sg1_FOR | ACCGTATTACAGAAATACTCTGAA |
| hBLM_sp_sg1_REV | AAACTTCAGAGTATTTCTGTAATA |
| hBMPR2_cpf1_sg1_FOR | AGATAGGATATGCAGGTTCTCGTGTCT |
| hBMPR2_cpf1_sg1_REV | AAAAAGACACGAGAACCTGCATATCCT |
| hBMPR2_sp_sg1_FOR | ACCGTATGCAGGTTCTCGTGTCTA |
| hBMPR2_sp_sg1_REV | AAACTAGACACGAGAACCTGCATA |
| hBTK_cpf1_sg1_FOR | AGATATCATCTCGACGGCCACGTCGTA |
| hBTK_cpf1_sg1_REV | AAAATACGACGTGGCCGTCGAGATGAT |
| hBTK_sp_sg1_FOR | ACCGTCTCGACGGCCACGTCGTAC |
| hBTK_sp_sg1_REV | AAACGTACGACGTGGCCGTCGAGA |
| hCHD2_cpf1_sg1_FOR | AGATCTGCAGATCGAGGAGACTGGCAG |
| hCHD2_cpf1_sg1_REV | AAAACTGCCAGTCTCCTCGATCTGCAG |
| hCHD2_sp_sg1_FOR | ACCGAGATCGAGGAGACTGGCAGA |
| hCHD2_sp_sg1_REV | AAACTCTGCCAGTCTCCTCGATCT |
| hCLN6_cpf1_sg1_FOR | AGATctccgcagCcGGcATTCCCTCTC |
| hCLN6_cpf1_sg1_REV | AAAAGAGAGGGAATGCCgGctgcggag |
| hCLN6_sp_sg1_FOR | ACCGagCcGGCATTCCCTCTCGAG |
| hCLN6_sp_sg1_REV | AAACCTCGAGAGGGAATGCCgGct |
| hGARS_cpf1_sg1_FOR | AGATTTTTGGATAGCGGAAGCCCTGAC |
| hGARS_cpf1_sg1_REV | AAAAGTCAGGGCTTCCGCTATCCAAAA |
| hGARS_sp_sg1_FOR | ACCGGGATAGCGGAAGCCCTGACC |
| hGARS_sp_sg1_REV | AAACGGTCAGGGCTTCCGCTATCC |
| hPAFAH1B1_cpf1_sg1_FOR | AGATAGGTGGGTCATGATAACTGGGTA |
| hPAFAH1B1_cpf1_sg1_REV | AAAATACCCAGTTATCATGACCCACCT |
| hPAFAH1B1_sp_sg1_FOR | ACCGGGTCATGATAACTGGGTACG |
| hPAFAH1B1_sp_sg1_REV | AAACCGTACCCAGTTATCATGACC |
| hPDE6C_cpf1_sg1_FOR | AGATACAGGAAGTTGCGGGTGGAGGTG |
| hPDE6C_cpf1_sg1_REV | AAAACACCTCCACCCGCAACTTCCTGT |
| hPDE6C_sp_sg1_FOR | ACCGAAGTTGCGGGTGGAGGTGCT |
| hPDE6C_sp_sg1_REV | AAACAGCACCTCCACCCGCAACTT |
| hPGM3_cpf1_sg1_FOR | AGATGGTGAAATGTcGGCACCATCCTG |
| hPGM3_cpf1_sg1_REV | AAAACAGGATGGTGCCgACATTTCACC |
| hPGM3_sp_sg1_FOR | ACCGAATGTcGGCACCATCCTGGG |
| hPGM3_sp_sg1_REV | AAACCCCAGGATGGTGCCgACATT |
| hPMS2_cpf1_sg1_FOR | AGATGGCAGAAGCAGGTAGTGATGGCC |
| hPMS2_cpf1_sg1_REV | AAAAGGCCATCACTACCTGCTTCTGCC |
| hPMS2_sp_sg1_FOR | ACCGCAGAAGCAGGTAGTGATGGC |
| hPMS2_sp_sg1_REV | AAACGCCATCACTACCTGCTTCTG |
| hSPAST_cpf1_sg1_FOR | AGATAAGCAGGACAGAAGGAGCAAGCT |
| hSPAST_cpf1_sg1_REV | AAAAAGCTTGCTCCTTCTGTCCTGCTT |
| hSPAST_sp_sg1_FOR | ACCGAGGACAGAAGGAGCAAGCTG |
| hSPAST_sp_sg1_REV | AAACCAGCTTGCTCCTTCTGTCCT |

**Supplementary Table 2: Oligos used for ABEmax-gRNA-expressing plasmid construction.**

| hBTK_ABE_sg1_FOR | ACCGGCCATCAAGATGATCAAAGA |
| --- | --- |
| hBTK_ABE_sg1_REV | AAACTCTTTGATCATCTTGATGGC |
| hCLN6_ABE_sg1_FOR | ACCGAATACCAGctgcggagcaaa |
| hCLN6_ABE_sg1_REV | AAACtttgctccgcagCTGGTATT |
| hPGM3_ABE_sg1_FOR | ACCGGGTGCCAACATTTCACCCAA |
| hPGM3_ABE_sg1_REV | AAACTTGGGTGAAATGTTGGCACC |

**Supplementary Table 3: gRNA target sequences and PCR primers for amplifying genomic DNA.**

| Name | Sequence(5' to 3') |
| --- | --- |
| crBLM | TTTCAGTATTACAGAAATACTCTGAAT |
| sgBLM | TATTACAGAAATACTCTGAATGG |
| hBLM_G2F | CTCTGAGGTGCTAACTTTGC |
| hBLM_G2R | GCACTTGCATTACGGGAACC |
| crBMPR2 | TTTGAGGATATGCAGGTTCTCGTGTCT |
| sgBMPR2 | TATGCAGGTTCTCGTGTCTAGGG |
| hBMPR2_G2F | TTTAGGATTTCCAAATGTGC |
| hBMPR2_G2R | GCACTACTTAACAGTTTTGG |
| crBTK | TTTGATCATCTcGAcGGCCACGTCGTA |
| sgBTK | TCTcGAcGGCCACGTCGTACTGG |
| hBTK_G1F | gcttagtctatcttcttgcctt |
| hBLM_G1R | ttatcttagcacttaggactacc |
| crCHD2 | TTTCCTGCAGATCGAGGAGACTGGCAG |
| sgCHD2 | AGATCGAGGAGACTGGCAGAGGG |
| hCHD2_G3F | CGAGAATCGCTTGACCCAAG |
| hCHD2_G3R | CCTATAACCAAGACATGGGA |
| crCLN6 | tttgctccgcagCcGGcATTCCCTCTC |
| sgCLN6 | agCcGGcATTCCCTCTCGAGTGG |
| hCLN6_G1F | ggaattgcatctgctctgtgtc |
| hCLN6_G1R | cttggggcatattttccatccc |
| crGARS | TTTATTTTGGATAGCGGAAGCCCTGAC |
| sgGARS | GGATAGCGGAAGCCCTGACCAGG |
| hGARS_G2F | TCCATTGACCTGCTTAGAGC |
| hGARS_G2R | GCAGTACACATTTCTAAGGC |
| crPAFAH1B1 | TTTAAGGTGGGTCATGATAACTGGGTA |
| sgPAFAH1B1 | GGTCATGATAACTGGGTACGTGG |
| hPAFAH1B1_G1F | TTACCCCCAAGTATTTTAGC |
| hPAFAH1B1_G1R | GCGTACATACCCAAGGAGGT |
| crPDE6C | TTTGACAGGAAGTTGCGGGTGGAGGTG |
| sgPDE6C | AAGTTGCGGGTGGAGGTGCTGGG |
| hPDE6C_G1F | CACTCTGCCTCAGGTAGTGC |
| hPDE6C_G1R | GCCCAACCCACTATCCCAAT |
| crPGM3 | TTTGGGTGAAATGTcGGCACCATCCTG |
| sgPGM3 | AATGTcGGCACCATCCTGGGAGG |
| hPGM3_G2F | ggtgtttattgttgaagctgc |
| hPGM3_G1R | aggcaggtagacatgcaactc |
| crPMS2 | TTTGGGCAGAAGCAGGTAGTGATGGCC |
| sgPMS2 | CAGAAGCAGGTAGTGATGGCCGG |
| hPMS2_G1F | GATCATTCTGTCAACAGTGC |
| hPMS2_G1R | GCATGCTTGTAATCCCACTA |
| crSPAST | TTTAAAGCAGGACAGAAGGAGCAAGCT |
| sgSPAST | AGGACAGAAGGAGCAAGCTGTGG |
| hSPAST_G1F | TAAGGATCAATACCATGGGC |
| hSPAST_G1R | GCCTGGGTAACAGAGGAAAG |
