# Supplementary material for: Comparison of cytosine base editors and development of the BEable-GPS database for targeting pathogenic SNVs
Source: Genome Biol. 2019 Oct 23;20:218. doi: 10.1186/s13059-019-1839-4 (PMC6806563; doi:10.1186/s13059-019-1839-4)
Supplement: Supplementary file 7 — Additional file 7. Review history. [file 13059_2019_1839_MOESM7_ESM.docx]

Review History

**First round of review**

**Reviewer 1**

**Are you able to assess all statistics in the manuscript, including the appropriateness of statistical tests used?**

yes

**Comments to author:**

The authors compared the activities of three base editors (BEs) at eight previously-reported target sites and eight human pathogenic sites and found that hA3AY130F-nCas9-eBE was most efficient for base editing. Then, the authors designed guide RNAs for targeting all human pathogenic point mutations using 10 different base editors. This guide RNA database was named the BEable-GPS database.  

Although the paper does not include breakthrough or surprising findings, I think that both the database of designed sgRNAs for the base editing of human pathogenic point mutations and the activity comparison results will be useful information for the genome editing community. Specific comments are as follows. 

Major comment
1. The title of the manuscript, "BEable-GPS database to compare base editors with different Cas proteins and cytidine deaminases", could be misleading. The main purpose of the webtool would be to guide base editor design for targeting pathogenic SNVs rather than to compare base editors with different Cas proteins and cytidine deaminases. Furthermore, "BEable-GPS" was not used for comparing base editor activities even in the current study. Thus, the title could be "BEable-GPS database as a tool for designing base editors that target pathogenic SNVs". If the authors want to incorporate the contents of Fig. 1 into the title, "Comparison of the activities of three base editors and base editor designing tools for targeting pathogenic SNVs" could be used. 

Minor comments 
1. What cell line was used for the experiments in Fig. 1d? This information should be described in the results section and/or figure legend. 
2. In Fig. S1, 3x free UGI should be separated from the BE construct. Currently, the 3x free UGI is depicted as linked to the BE construct, which could be misleading to readers.

**Reviewer 2**

**Are you able to assess all statistics in the manuscript, including the appropriateness of statistical tests used?**

yes

**Comments to author:**

Wang and coworkers set out to compare the editing efficiency and accuracy of three different C:T base editors. The authors also develop a database for users to determine which base editor would be best suited for the user's application. Additionally, Wang et al. have proposed to rename all the base editors based on the combination of Cas and deaminase moieties contained in the base editor.
The proposed nomenclature for the proteins, e.g., BE3 to rA1-nCas9-BE, is confusing and complex. Is there really a need to change the names? Renaming the proteins should be considered at a conference or an opinion/editorial article.
The authors only compared 3 out of 10 base editors for efficiency and accuracy. 
The manuscript reports using the base editors for generating pathogenic mutations, not correcting them. While having cell lines/animal models carrying pathogenic SNPs are important to study disease, these mutations can easily be generated via HDR using CRISPR/Cas and a donor template. The base editors should be tested on their ability to correct a pathogenic SNP rather than creating one. 
The BE-able GPS database is a terrific idea and will be a great resource. However, the authors did not show any validity of the database's profiling. It would be nice to see if the base editor chosen by the database is superior or at least equivalent to other base editors. 
The current manuscript is not yet mature enough for Genome Biology or should be submitted elsewhere.

**Authors Response**

**Point-by-point responses to the reviewers’ comments:**

*We like to thank reviewers for their insightful and helpful suggestions that have guided us to improve this manuscript. In this revised manuscript, we have addressed all points raised by the reviewers with revisions and the addition of new analyses and discussion. Specifically, we have included cases to show that BEs can be used to correct pathogenic SNVs. Please find our point-by-point responses below.* 

Reviewer #1: The authors compared the activities of three base editors (BEs) at eight previously-reported target sites and eight human pathogenic sites and found that hA3AY130F-nCas9-eBE was most efficient for base editing. Then, the authors designed guide RNAs for targeting all human pathogenic point mutations using 10 different base editors. This guide RNA database was named the BEable-GPS database. 
Although the paper does not include breakthrough or surprising findings, I think that both the database of designed sgRNAs for the base editing of human pathogenic point mutations and the activity comparison results will be useful information for the genome editing community. 
*We thank reviewer #1 for his/her general supports.* 

Specific comments are as follows. 
Major comment 
1. The title of the manuscript, "BEable-GPS database to compare base editors with different Cas proteins and cytidine deaminases", could be misleading. The main purpose of the webtool would be to guide base editor design for targeting pathogenic SNVs rather than to compare base editors with different Cas proteins and cytidine deaminases. Furthermore, "BEable-GPS" was not used for comparing base editor activities even in the current study. Thus, the title could be "BEable-GPS database as a tool for designing base editors that target pathogenic SNVs". If the authors want to incorporate the contents of Fig. 1 into the title, "Comparison of the activities of three base editors and base editor designing tools for targeting pathogenic SNVs" could be used. 
*Thanks for the suggestion. We have now changed the title to “Comparison of cytosine base editors and BEable-GPS database for targeting pathogenic SNVs” accordingly.* 

Minor comments 
1. What cell line was used for the experiments in Fig. 1d? This information should be described in the results section and/or figure legend. 
*Thanks. 293FT cells were used for the experiments performed in Fig.1 and we have now added the cell line description in the revised figure legend of Fig. 1d and 1e.* 

2. In Fig. S1, 3x free UGI should be separated from the BE construct. Currently, the 3x free UGI is depicted as linked to the BE construct, which could be misleading to readers. 
*Thanks. DNA sequences for three copies of 2A-UGI are directly linked to sequences for Cas protein and cytidine deaminase in BE constructs. After transcription and translation, these 2A-UGI will be then expressed as free UGI as this reviewer suggested. The schematic diagram in originally submitted version is to illustrate the design of BE expression vectors, and their sequences are shown to link with those for Cas protein and cytidine deaminase. In the revised manuscript, to avoid any possible misunderstanding, we have removed the redundant schematic diagram, and instead have added the related description in the revised Fig. 2a to show BEs with or without three copies of free UGIs.* 


Reviewer #2: Wang and coworkers set out to compare the editing efficiency and accuracy of three different C:T base editors. The authors also develop a database for users to determine which base editor would be best suited for the user's application. Additionally, Wang et al. have proposed to rename all the base editors based on the combination of Cas and deaminase moieties contained in the base editor. 
The proposed nomenclature for the proteins, e.g., BE3 to rA1-nCas9-BE, is confusing and complex. Is there really a need to change the names? Renaming the proteins should be considered at a conference or an opinion/editorial article. 
*Thanks for the suggestion. We have now removed the nomenclature part of this manuscript to avoid any possible confusion.* 

The authors only compared 3 out of 10 base editors for efficiency and accuracy. 
Thanks. In the revised manuscript (revised Fig. 1), we have included two additional BEs, BE3, a commonly used BE, and BE4max, one of the most efficient BEs, for comparison of their efficiencies and product purity in C-to-T base editing. We think that these five BEs well represent most of currently available cytosine base editors. In the revised manuscript, we have now included 20 BEs to construct the BEable-GPS database. 

*The manuscript reports using the base editors for generating pathogenic mutations, not correcting them. While having cell lines/animal models carrying pathogenic SNPs are important to study disease, these mutations can easily be generated via HDR using CRISPR/Cas and a donor template. The base editors should be tested on their ability to correct a pathogenic SNP rather than creating one. 
Thanks for this inspiring suggestion. We think it is a great idea to show the base editing efficiency and product purity in correcting pathogenic mutations. In the revised manuscript, we have applied ABEmax to first generate three cell lines each containing a distinct pathogenic A-to-G/T-to-C SNV (Revised Fig. S3) and then have compared the correction of these SNVs with five BEs (Revised Fig. 1e). The correction efficiencies of these five BEs are similar to their efficiencies to generate C-to-T pathogenic SNVs (Revised Fig. 1d). 

The BE-able GPS database is a terrific idea and will be a great resource. However, the authors did not show any validity of the database's profiling. It would be nice to see if the base editor chosen by the database is superior or at least equivalent to other base editors. 
The current manuscript is not yet mature enough for Genome Biology or should be submitted elsewhere. 
Thanks for the comments on the development of BEable-GPS database. In this revised manuscript, we have included the correction of three pathogenic SNVs by BEs. Since suitable BEs and their guide RNA sequences for correcting pathogenic SNVs are all available in BEable-GPS, the correction experiment in revised Fig. 1e shows at least a set of validity of BEable-GPS. 

Of note, the genomic locations of known pathogenic SNVs have been determined at the ClinVar database, and thus their available PAM sequences and accessibilities to BEs are fixed. BEable-GPS is only used to summarize all possible conditions for BE correction. Also, the correction efficiency and accuracy of each pathogenic SNP by BEs should be carefully examined before any therapy-related test.*
